# Neonatal Eating Assessment Tool - Mixed Breastfeeding and Bottle-Feeding (NeoEAT - Mixed Feeding): factor analysis and psychometric properties

**DOI:** 10.1186/s40748-019-0107-7

**Published:** 2019-07-31

**Authors:** Britt Frisk Pados, Suzanne M. Thoyre, Kara Galer

**Affiliations:** 1 Boston College William F. Connell School of Nursing, Maloney 268, Chestnut Hill, MA 02467 USA; 2 University of North Carolina at Chapel Hill School of Nursing, Carrington Hall, Chapel Hill, NC 27599 USA

**Keywords:** Bottle feeding, Breast feeding, Feeding behavior, Surveys and questionnaires, Psychometrics, Patient reported outcome measures

## Abstract

**Background:**

Early identification of feeding difficulty in infancy is critical to supporting breastfeeding and ensuring optimal nutrition for brain development. The Neonatal Eating Assessment Tool (NeoEAT) is a parent-report assessment that currently has two versions: NeoEAT – Breastfeeding and NeoEAT – Bottle-feeding for use in breast and bottle-fed infants, respectively. There are currently no valid and reliable parent-report measures to assess feeding through a combination of both breast and bottle delivery. The purpose of this study was to conduct a factor analysis and test the psychometric properties of a new measure, the NeoEAT – Mixed Breastfeeding and Bottle-Feeding (NeoEAT – Mixed Feeding), including internal consistency reliability, test-retest reliability, construct validity and known-groups validity.

**Methods:**

Parents of infants younger than 7 months who had fed by both bottle and breast in the previous 7 days were invited to participate. Internal consistency reliability was tested using Cronbach’s α. Test-retest reliability was tested between scores on the NeoEAT – Mixed Feeding completed 2 weeks apart. Construct validity was tested using correlations between the NeoEAT – Mixed-Feeding, the Infant Gastroesophageal Reflux Questionnaire - Revised (I-GERQ-R), and the Infant Gastrointestinal Symptoms Questionnaire (IGSQ). Known-groups validation was tested between healthy infants and infants with feeding problems.

**Results:**

A total of 608 parents participated. Exploratory factor analysis revealed a 68-item scale with 5 sub-scales. Internal consistency reliability (Cronbach’s α = .88) and test-retest reliability (*r* = 0.91; *p* < .001) were both acceptable. Construct validity was demonstrated through correlations with the I-GERQ-R (*r* = 0.57; *p* < .001) and IGSQ (*r* = 0.5; *p* < .001). Infants with feeding problems scored significantly higher on the NeoEAT – Mixed Feeding, indicating more problematic feeding symptoms, than infants without feeding problems (*p* < .001), supporting known-groups validity.

**Conclusions:**

The NeoEAT – Mixed Feeding is a 68-item parent-reported measure of breast- and bottle-feeding behavior for infants less than 7 months old that now has evidence of validity and reliability for use in clinical practice and research. The NeoEAT – Mixed Feeding can be used to identify infants with problematic feeding, guide referral decisions, and evaluate response to interventions.

## Background

The World Health Organization Global Nutrition Target is for 50% of infants worldwide to exclusively breastfeed for the first 6 months of life by the year 2025 [1]. Breastfeeding rates in the United States have been increasing in recent years, but the most recently available data suggest that only 24.9% of infants in the United States are exclusively breastfed at 6 months [2]. While not all mothers desire to meet this goal of exclusive breastfeeding (or provision of human milk) through 6 months, one study found that 60% of women in the United States reported that they were not able to meet their desired goals for breastfeeding [3]. Reasons for early cessation of breastfeeding are complex, but those who did not meet their desired goals for breastfeeding were significantly more likely to cite infant feeding difficulty, specifically sucking or latching difficulty, as the reason for early cessation compared to women who met their breastfeeding goals [3]. Up to 53.7% of mothers who attempt to breastfeed have attributed their discontinuation of breastfeeding in the first month to infant feeding difficulties [4, 5].

Early identification of feeding difficulty in infancy is critical for supporting continuation of breastfeeding and ensuring optimal nutrition for brain development. Problematic feeding can be challenging to diagnose given the variation and nuance in symptom presentation [6]. As a result, feeding assessments have historically focused on feeding outcomes (e.g. volume of intake, changes in vital signs) as measures of skill, with interventions applied generically [7]. Assessments which focus on infant behavior throughout the feeding are critical for identifying individual problem areas and implementing personalized strategies to optimize nutrition and oral feeding skill development [7]. While clinician assessments are a critical component to the overall assessment of oral feeding, clinicians vary in their knowledge about infant feeding and parent-reported assessments can provide an objective means of guiding the clinician in their decision-making.

Several tools have been published for the purpose of assessing feeding behaviors in infants who are either breastfeeding or bottle-feeding [6, 8, 9]. These tools include content specific to the assessment of breastfeeding or bottle-feeding behaviors, but infants who receive a combination of both feeding methods may present with problematic feeding behaviors not accounted for in existing tools. For example, the infant who is being fed with a combination of methods must be willing and able to manage differences in flow rates between the breast and bottle and alter their oral mechanics to latch on to both a soft, pliable breast and a more firm, structured bottle nipple. Therefore, a valid and reliable assessment is needed for infants receiving mixed breastfeeding and bottle-feeding.

### Literature review

A systematic review was conducted in 2015 to evaluate measures available for the assessment of feeding in young infants [6]. As of June 2015, two assessment tools were identified that could be used for infants who were both breast- and bottle-feeding: the Early Feeding Skills (EFS) assessment and the Neonatal Oral Motor Assessment Scale (NOMAS) [6]. Both of these assessment tools are clinician-reported assessments, meaning that a clinician answers the questions and the questions are written for those with advanced knowledge in infant feeding.

An updated review was conducted to include literature from June 1st, 2015 through February 1st, 2019 to determine whether a parent-report assessment tool was currently available for evaluating feeding when an infant was both breast- and bottle-feeding. The search strategy replicated the strategy used in the 2015 review. The terms used for the search were “infant feeding” and “assessment tool.” The search was limited to English language, human, and full text. Both articles and textbooks were included.

The literature was reviewed by the research team for presentation of new assessment tools, use of existing tools, or reference to existing tools. Assessment tools were excluded if they were intended only for infants older than 7 months, were intended for assessment of solid food feeding (e.g., pureed baby food), or were intended to assess a construct other than the infant’s behavior during feeding (e.g., parent-infant interaction, breastfeeding self-efficacy, feeding readiness). Once tools were identified, a secondary specific search of tools by name via PubMed and CINAHL was conducted to identify additional literature on that specific tool. Since the intent of this review was to assess evidence for both clinical practice and research, assessment tools were further excluded if they lacked sufficient published literature to evaluate the tool, if the target population was limited to a specific diagnosis, or if the tool was intended for research only (i.e., not intended for clinical use).

**Fig. 1:**
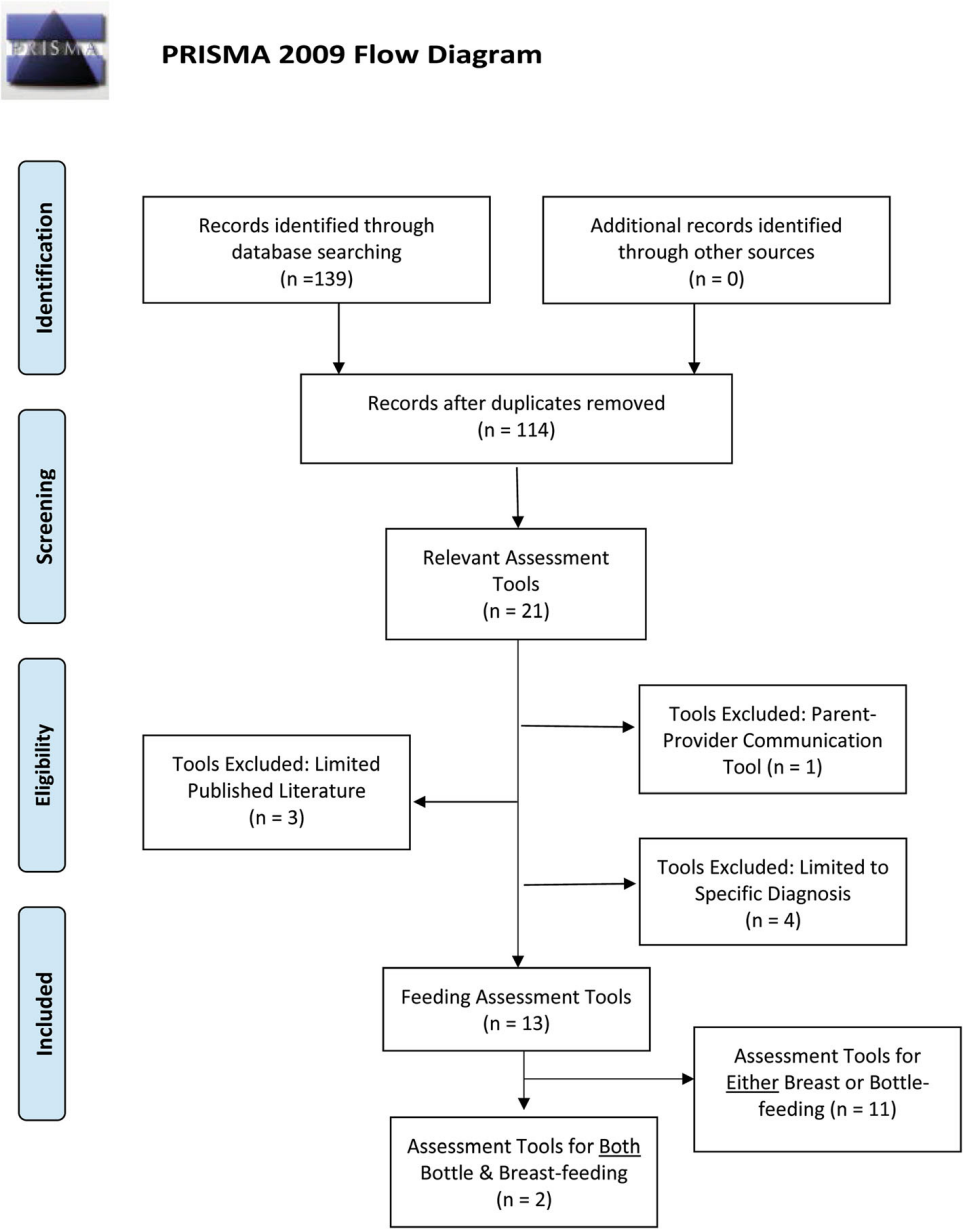
Preferred Reporting items for Systematic Reviews and Meta-Analyses (PRIMSA) diagram of literature search results. Diagram retrieved from: http://prisma-statement.org/PRISMAStatement/FlowDiagram.aspx

The initial search of databases resulted in 114 unique articles and texts for review (Fig. 1). From this literature, 21 relevant tools were identified that met inclusion criteria. Three of these tools were excluded because they did not have adequate published literature for evaluation of the tool: B-R-E-A-S-T-Feed Observation Form [10], Infant Nipple Feeding Assessment and Communication Tool [11], and the Via Christi Breastfeeding Assessment [12]. Four additional tools were excluded because their use is limited to specific diagnoses: the Feeding Checklist (infants with non-organic failure to thrive) [13], the Infant Malnutrition and Feeding Checklist for Congenital Heart Disease (infants with congenital heart disease) [14], the Nutrition and Feeding Risk Identification Tool (infants in Early Intervention care) [15] and the Neonatal Eating Outcome (NEO) Assessment (premature infants) [16]. The Infant and Child Feeding Questionnaire (ICFQ) was excluded because it is an anticipatory guidance and engagement tool intended to facilitate effective conversations between caregivers and providers, but is not intended to be used as an assessment tool for the purposes of clinical decision-making [17].

Of the 13 remaining assessment tools, 10 were excluded because they were designed to assess exclusively breastfed infants. The breastfeeding-specific assessment tools were the: Breastfeeding Evaluation and Education Tool [18], Bristol Breastfeeding Assessment Tool [19], Infant Breastfeeding Assessment Tool [20], LATCH [21], Mother-Baby Assessment [22], Mother-Infant Breastfeeding Progress Tool [23], Neonatal Eating Assessment Tool – Breastfeeding [9], Potential Early Breastfeeding Problem Tool [24], Premature Infant Breastfeeding Behavior Scale [25], and Systematic Assessment of the Infant at Breast [26]. The psychometric properties of these tools are described in another recent publication [9]. Of the remaining three assessment tools, one was intended for exclusively bottle-fed infants: the Neonatal Eating Assessment Tool – Bottle-feeding [8].

Similar to the findings of the review completed in 2015, the only two tools available for the assessment of infants feeding by both bottle and breast were the EFS [27, 28] and the NOMAS [29–35]. The psychometric properties of the EFS and NOMAS are presented on Table 1. With recent updates to these tools, these tools now have adequate psychometric properties, but they are both clinician-reported instruments and neither of these tools specifically evaluates an infant’s ability or willingness to move between both breast- and bottle-feeding methods. Clinician-report assessments have an essential role in the overall clinical assessment of an infant, but parent-report assessments are complementary in a number of ways. Parent-report assessments do not require training or specialty knowledge, and therefore can be used more broadly across different healthcare settings. Additionally, parents are in a unique position to report on behaviors seen over the course of many days, which may be different from a short clinical assessment that may or may not be timed well with a feeding.

**Table 1:** Psychometric Properties of Currently Available Tools for the Assessment of Infants who are Both Breast- and Bottle-Feeding

Tool (Author)	Purpose & intended user	Target population	Items – number & constructs	Reliability			Validity	
				Internal consistency	Inter-rater	Test-retest	Content	Construct
Early Feeding Skills (EFS) [27, 28]	Assess oral feeding skills; Clinician-reported (Training available, but not required)	Preterm and term infants birth to 52 weeks post-menstrual age.	19 items on revised version; Respiratory Regulation, Oral-Motor Function, Swallowing Coordination, Engagement, Physiologic Stability	+	+	NR	*	+
				Acceptable internal consistency reliability (Cronbach’s α = .81) [28]. Inter-rater reliability reported as acceptable between trained clinicians.			* Item generation consistent with content validity, but not tested [27]. Construct validity with the Infant Drive Feeding Scale – Quality (*r* = −.18- - .73; *p* < .05) [28].	
Neonatal Oral Motor Assessment Scale (NOMAS) [29]	Describe disorganized and dysfunctional sucking patterns; Clinician-reported (Training Required)	Preterm infants and those with complex medical conditions.	28 items; Jaw and tongue movement and function	+	+	+/−	NR	+/−
				Internal consistency reliability acceptable [30]. Inter-rater reliability acceptable after scoring system change (k = .78–.9) [31]. Test-retest inconsistent (k = .33–.94 and *r* = .23–.57) [32, 33].			Inconsistent evidence of construct validity with related constructs across studies [32, 34, 35].	

+ = Acceptable reliability/ validity defined as Cronbach’s α > .7 [36], Cohen’s k > .6, ICC > .75, percent agreement > 80% [37], or correlation coefficient (*r*) > .4 [38]; − = Unacceptable reliability/validity by these definitions; +/− = Mixed findings; * = Description of item generation is consistent with content validity, but no formal testing conducted; *NR* = Not Reported

The review of the current literature determined that there were currently no valid and reliable parent-report measures available to assess feeding when an infant was both breast- and bottle-feeding. The Neonatal Eating Assessment Tool - Mixed Breastfeeding and Bottle-Feeding (NeoEAT – Mixed Feeding) was designed to fulfill this need for infants aged less than 7 months. Items on the NeoEAT – Mixed Feeding were developed and content validated [39] according to instrument development guidelines [40].

## Methods

### Aims

The aim of this study was to determine the factor structure of the NeoEAT - Mixed Feeding and to assess its psychometric properties, including internal consistency reliability, test-retest reliability, construct validity and known-groups validity.

### Design

This was a descriptive, cross-sectional, instrument development study.

### Setting

This study was conducted using online surveys through the Qualtrics survey platform. Parents were recruited for participation in this study through a variety of methods, including recruitment through Qualtrics respondent panels; a pediatric primary care clinic, pediatric feeding and swallowing clinic, and infants who had been discharged from the Neonatal Intensive Care Unit at North Carolina Children’s Hospital; ResearchMatch.com, a national health volunteer registry supported by the National Institutes of Health and the Clinical Translational Science Award (CTSA) program; Join the Conquest, a health volunteer registry through the CTSA at the University of North Carolina at Chapel Hill; a registry of parents of children with problematic feeding maintained by the investigative team; online parent support groups; and an email sent to faculty, staff, and students at the University of North Carolina at Chapel Hill.

### Sample

To be eligible to participate in the study, parents had to be at least 18 years old and have an infant less than 7 months old who had been fed by both breast and bottle in the previous 7 days. Parents, for the purposes of this study, were defined as primary caregivers who were familiar with the child’s feeding and are referred to as parents throughout this manuscript. Participants had to have access to the internet in order to complete the survey and had to self-report as being able to read English. Only one parent was allowed to participate per family. If a parent had more than one infant less than 7 months old, they were asked to report on a single infant. The goal was to have parents report on a heterogeneous sample of infants, so infants were not excluded for any medical reasons, but the infant did have to be fed by mouth in the past 7 days, so exclusively tube-fed infants were excluded. The target sample size for the factor analysis was 5–10 participants per item [41]. With 89 items on the original NeoEAT – Mixed Feeding, the target sample for the factor analysis was a minimum of 445 participants.

### Measures

#### NeoEAT - Mixed Feeding

The NeoEAT – Mixed Feeding is an 89-item parent-report measure of symptoms of problematic feeding with items that are relevant to breastfeeding, bottle-feeding, and the infant’s ability or willingness to manage changes between breast- and bottle-feeding. Items on the NeoEAT – Mixed Feeding were developed and content validated with both parents (*N* = 16) and clinicians (*N* = 9) [39]. Items on the NeoEAT – Mixed Feeding are prefaced with the phrase “My infant …” followed by a short phrase stating a behavior or symptom that would be observable by a parent with little feeding experience. Items are written at a less than 6th grade reading level [39], which is consistent with recommendations for health-related materials [42]. Response options on the NeoEAT – Mixed are on a 6-point Likert scale from Never to Always. Scores are assigned such that higher scores indicate more symptoms of problematic feeding. Positively worded items are reverse-scored to maintain consistency across the items, with higher scores indicating more problematic symptoms. The possible range of scores for the 89-item NeoEAT – Mixed Feeding was 0 to 445. The NeoEAT – Mixed Feeding is intended for infants less than 7 months who are obtaining the majority of their nutrition from liquid-based feeding (i.e., human milk and/or infant formula). The questionnaire takes approximately 5 to 10 min to complete.

#### Infant gastroesophageal reflux questionnaire – revised (IGERQ-R)

The IGERQ-R is a 12-item caregiver-report measure of gastroesophageal reflux-related symptoms in infants over the previous 7 days [43–45]. The IGERQ-R was chosen as a measure to test convergent validity because it is a parent-report assessment of a construct measured by the NeoEAT – Mixed Feeding, specifically symptoms related to gastroesophageal reflux. The tool is scored on a scale from 0 to 42, with a higher score indicating more symptoms of gastroesophageal reflux. The tool has been validated for use as both an evaluative and diagnostic instrument. Psychometric testing supports the diagnostic capability of the tool, demonstrating its ability to discriminate infants meeting the criteria for GERD diagnosis from those who do not, as well as between infants with mild, moderate and severe disease [43]. Psychometric properties also support its responsiveness to change in GERD symptoms over time, making it a valuable tool for monitoring treatment in clinical practice and evaluating outcomes in clinical trials [43]. The tool was content validated with both caregivers and physicians. Internal consistency reliability (Cronbach α = 0.86–0.87), test-retest reliability (intraclass correlation coefficient = 0.85), and construct validity were all found to be acceptable [43].

#### Infant gastrointestinal symptoms questionnaire (IGSQ)

The IGSQ is a 13-item parent-report questionnaire about the frequency and severity of gastrointestinal symptoms in infants in the previous 7 days [46]. The IGSQ was chosen as a parent-report measure to test convergent validity between the IGSQ and symptoms of gastrointestinal distress as measured by the NeoEAT – Mixed Feeding. The tool is scored on a scale from 13 to 65, with a higher score indicating more symptoms of gastrointestinal distress [46]. The tool is useful for clinical research on feeding tolerance and identification of infants with gastrointestinal distress. The tool has evidence of acceptable internal consistency reliability (Cronbach’s α = 0.72) and test-retest reliability (*r* = 0.69) [46]. Known-groups validity was supported with significant differences in scores between infants with and without parent-reported feeding problems. The tool has also shown to be sensitive to differences between human milk–fed and formula-fed infants [46].

### Procedures

Parents who agreed to participate in the research study were asked to complete a survey that included the NeoEAT – Mixed Feeding, IGERQ-R, IGSQ, a series of questions about their child’s health and feeding, and questions to describe the respondent and their family. Parents were given 2 weeks to complete the survey. During this time, two reminder emails were sent to those who had not yet finished. Parents who completed the initial survey were offered a $10 gift card. The first 20% of the sample were asked whether they would be interested in completing a second survey 2 weeks later for the purpose of evaluating test-retest reliability. The second survey only included the NeoEAT – Mixed Feeding and therefore was much shorter than the initial survey. Parents were only given 1 week to complete the second survey so that the test-retest surveys were 2–3 weeks apart. Parents who completed the retest survey were offered an additional $5 gift card.

Given the potential threats to validity with online survey research, multiple strategies were employed to ensure the validity of the data used for analysis. Participants recruited through North Carolina Children’s Hospital were identified as eligible through medical record review. All other participants entered the survey through a two-step entry process, allowing for only a single response per individual. Response times to the survey were monitored and respondents who completed the survey in less than one third of the median completion time (defined by the first 10% of the sample), were removed from the survey as their responses were deemed to be too fast to reflect thoughtful and accurate data. Attention-check and verification questions were placed throughout the survey to identify careless or fraudulent respondents. Data were monitored closely and cleaned thoroughly prior to analysis.

### Data analysis

Data analyses were conducted using IBM SPSS Statistics 24. Cases with > 10% missing data for the NeoEAT – Mixed Feeding were excluded from the overall analysis. Cases with > 10% data on the IGSQ, IGERQ-R or retest survey were excluded for each of those analyses separately. A missing data analysis was conducted prior to other statistical analyses. For all statistical tests, a *p*-value of .05 was defined as statistically significant.

#### Item analysis

First, inter-item correlations were calculated using Pearson’s product-moment correlation. The correlation matrix was evaluated for item-item correlations > .8 (indicating the items were measuring the same construct) and items that failed to correlate with any other item at > .3 (indicating the item may be measuring an unrelated construct) [47]. When two items were correlated at > .8, one of the items was chosen for removal. When an item failed to correlate with any other item at > .3, it was removed.

#### Factor analysis

Exploratory factor analysis was conducted using principal components analysis with varimax rotation. Procedures for factor analysis followed accepted guidelines for for health-related instrument development [47]. The Kaiser-Meyer-Olkin (KMO) statistic and Bartlett’s test of sphericity were evaluated as a measure of sample adequacy for factoring. Prior to further exploration of the factor analysis results, communalities of the items were reviewed and items with communalities < .5 were removed. The factor analysis was then repeated. Initially, factor extraction was based on an eigenvalue of greater than one, a method that ensures that each factor accounts for a considerable share of the total variance of the items; this method, however, can over- or under-estimate the correct number of factors [47]. The scree plot, which plots the factors against their eigenvalues in decreasing order, was then examined to determine whether a more parsimonious factor solution could be supported [47].

Using the scree plot and the number of factors represented around the bend in the curve, exploratory factor analysis was conducted forcing different factor solutions. Multiple factor solution options were explored, taking into account total variance explained, number of cross-loading items, and conceptual clarity of the factors [47]. Items that cross-loaded at > .3 on two factors were considered for movement to another factor based on the conceptual fit. Items that failed to load on any factor at > .3 were identified for removal. After final placement of items within the factors, factor names were assigned based on the concepts measured by the items within the factor; more weight was given to the most highly loaded items within each factor for naming purposes. After names were assigned to the factors, they were referred to as subscales.

#### Internal consistency reliability

First, internal consistency reliability was calculated within each subscale using Cronbach’s α. Acceptable Cronbach’s α is defined as greater than .7 [36]. Within each subscale, each item was evaluated for whether the subscale Cronbach’s α would increase significantly if the item were deleted. If removing an item would cause the Cronbach’s α for the subscale to change from being unacceptable to acceptable, the item was removed. Item-total correlations were evaluated as well with the target item-total correlation being greater than .3 [47]. After decisions were made about removing items within each subscale, the Cronbach’s α for the full scale was calculated.

#### Temporal stability

To evaluate stability of the measure over time, test-retest reliability was conducted between NeoEAT – Mixed Feeding scores that were collected from the same parent about the same infant 2–3 weeks apart. Bivariate correlations were calculated using Pearson’s product moment correlation (*r,* two-tailed) between the NeoEAT – Mixed Feeding scores in the initial survey with the NeoEAT – Mixed Feeding scores in the retest survey. Correlations were calculated between each subscale score as well as the total score. Because missing data would distort the subscale and/or total score and alter the test-retest reliability, cases with any missing data within each subscale were excluded from that subscale analysis and cases with any missing data at all were excluded from the NeoEAT – Mixed Feeding total test-retest reliability analysis.

#### Convergent validity

To evaluate convergent validity, scores on the NeoEAT – Mixed Feeding were evaluated for congruency with two other parent-report measures of related constructs: the IGERQ-R and the IGSQ. Bivariate correlations were calculated using Pearson’s product moment correlation (*r,* two-tailed) between the NeoEAT – Mixed Feeding scores (total and subscale scores), the IGERQ-R sum score, and the IGSQ sum score.

#### Known-groups validity

Known-groups validity was tested by comparing NeoEAT – Mixed Feeding total score and subscale scores between two groups that represented a subset of the sample from the factor analysis: 1) healthy infants with no feeding concerns and 2) infants with problematic feeding. To be included in the group of health infants with no feeding concerns, the parent had to report that the infant was born full-term, there were no feeding concerns, the infant did not take a prescription medication regularly, and did not have any of the following conditions: genetic disorder, congenital heart defect, developmental delay, or structural abnormality of the face, mouth, or gastrointestinal tract. To be included in the group of infants with problematic feeding, the parent had to report that either they thought the infant had a feeding problem, the infant had been diagnosed by a healthcare provider with a feeding problem, and/or the infant had a feeding tube. Data on infants who did not clearly fall into either of these categories were excluded from this analysis. Independent samples t-test was conducted comparing the two groups of infants for the NeoEAT – Mixed total score and all subscale scores.

## Results

### Sample

There were 608 parents who completed the survey, which exceeded the minimum target sample for factor analysis of 445. There were no missing data, so all 608 cases were included. The majority of participants were located in the United States (*n* = 599). Outside of the United States, there were participants from Australia (*n* = 1), Canada (*n* = 3), Malaysia (*n* = 1), Mexico (*n* = 1), and the United Kingdom of Great Britain and Northern Ireland (*n* = 2). Within the United States, there were participants from 44 states. The distribution of the infant sample by sex and corrected gestational age is reported on Table 2. The characteristics of the parent respondents and their families are reported on Table 3. The infants included in the sample were both healthy, full-term infants, and infants with a variety of health-related conditions (Table 3). A subset of the total sample also completed the IGSQ (*n* = 363), IGERQ-R (*n* = 601), and the retest survey 2 weeks after the first (*n* = 53).

**Table 2:** Summary of Sex and Age Distribution of Infant Sample

Corrected age	Sex		Total
	Male	Female	
0–2 months	87	89	176 (28.9%)
2–4 months	75	100	175 (28.8%)
4–6 months	91	92	183 (30.1%)
6–7 months	34	40	74 (12.2%)
Total	287 (47.2%)	321 (52.8%)	608

Corrected Age was calculated as the infant’s age on the date of survey completion, adjusting for preterm birth by subtracting the number of weeks the infant was born preterm from current age if the infant was born prior to 37 weeks post-menstrual age

**Table 3:** Descriptive statistics for respondents and their infants

Variable	Frequency (*n*)	Percent
Relationship to Infant (*n* = 608)		
Mother or Mother-Figure	575	94.6%
Father or Father-Figure	28	4.6%
Other Primary Caregiver	5	0.8%
Child Race/Ethnicity (*n* = 607)		
White	399	65.7%
Hispanic	43	7.1%
Black	35	5.8%
Asian	24	4.0%
More than one race	90	14.8%
Other	10	1.6%
Parent Highest Education (*n* = 608)		
High School degree or less	138	22.7%
Technical School/Community College	62	10.2%
College/University	206	33.9%
Graduate School	202	33.2%
Household Income (*n* = 605)		
< $20,000	49	8.1%
$20,000 – 39,999	112	18.5%
$40,000 – 59,999	102	16.9%
$60,000 – 79,999	92	15.2%
$80,000 – 99,999	57	9.4%
> $100,000	193	31.9%
Family Type (*n* = 608)		
Two Parent Family	550	90.5%
Single Parent Family	46	7.6%
Other	12	2.0%
Select Infant Conditions (*n* = 608)^a^		
Diagnosed Feeding Problem	38	6.3%
Current Feeding Tube	10	1.6%
Preterm Birth	67	11.0%
Structural Abnormality	18	3.0%
Congenital Heart Disease	18	3.0%
Genetic Disorder	5	0.8%

^a^Multiple conditions could be selected

### Item analysis

There were initially 89 items on the NeoEAT – Mixed Feeding. Evaluation of inter-item correlations identified 11 items for removal based on item-item correlation > .8. Additionally, four items were removed because they failed to correlate with any other item at > .3. After this process, 74 items remained.

### Factor analysis

Exploratory factor analysis with 74 items revealed a Kaiser-Meyer-Olkin (KMO) statistic of .888 and Bartlett’s test of sphericity was significant (*p* < .001), which indicated that the sample size was adequate for factor analysis [48, 49]. Three items were identified after the initial factor analysis as having communalities < .5, so these three items were removed. The factor analysis was then repeated with 71 items. Using an eigenvalue of greater than one, 18 factors were initially extracted, which explained 64.9% of the total variance. Upon examination of the scree plot, it was determined that a factor solution between four and six factors would be more appropriate and parsimonious. The four-, five-, and six-factor solutions were considered. There was a considerable loss of variance explained between the four and five factor solution, so the four-factor solution was deemed unacceptable. One item failed to load on any factor at > .3 in the five- and six-factor solution, so this item was removed. In the six-factor solution with 70 items, the sixth factor did not hold together conceptually, so the five-factor solution was identified as most appropriate. In the five-factor solution, one additional item failed to load at > .3 on any factor and one item had a very low, as well as negative, loading (−.308) and did not fit conceptually with the other items in the factor; both of these items were removed. The final solution was a five-factor solution with 68 items, which explained 40.67% of the total variance. The final placement of items within the five-factor solution are reported on Table 4. The factors were assigned the following names: *Gastrointestinal Tract Function* (27 items), *Infant Regulation* (11 items), *Energy & Physiologic Stability* (13 items), *Sensory Responsiveness* (7 items), and *Feeding Flexibility* (10 items).

**Table 4:** Final Item Placements and Factor Loadings for Principal Component Analysis with Varimax Rotation of the NeoEAT – Mixed Feeding

Subscale	NeoEAT – mixed feeding item (All items begin with “My baby …”)	Factor loadings
*Gastrointestinal Tract Function* 27 items Cronbach’s α .91	seems uncomfortable after feeding.	.68
	spits up in between feedings.	.63
	chokes or coughs during eating.	.62
	is uncomfortable if laid flat after eating.	.60
	becomes stiff/rigid during or after eating.	.59
	throws up in between feedings.	.59
	coughs in between feedings.	.58
	throws up during feeding.	.58
	spits up during feeding.	.57
	is very gassy.	.56
	becomes upset during feeding (whines, cries, gets fussy).	.56
	sounds gurgly or like they need to cough or clear their throat during or after eating.	.56
	coughs or chokes on saliva/spit when not eating.	.55
	arches back during or after eating.	.53
	gags in between feedings when there is nothing in his/her mouth.	.49
	gets a bloated (big or hard) tummy after eating.	.49
	needs to be burped more than once before the end of feeding.	.49
	tilts head back during or after eating.	.48
	gets a stuffy nose when eating.	.45
	gulps when eating (swallows loudly).	.44
	gets the hiccups.	.43
	drools milk out of the side of the mouth when feeding.	.40
	gets watery eyes when eating.	.39
	gets red color around eyes or face when eating.	.39
	gags on a pacifier or toys put in mouth.	.39
	gags on the bottle nipple.	.33
	turns red in face, may cry with stooling/pooping.	.38
*Infant Regulation* 11 items Cronbach’s α .86	eats enough to have at least 5 wet diapers per day (24 h).	.88
	is satisfied after eating.	.86
	is easy to console when upset (for example, stops crying when held or offered a pacifier).	.80
	roots when hungry (for example, sucks on fist, smacks lips, looks for breast/bottle).	.79
	is calm and relaxed when eating.	.77
	lets me know when he/she is done eating.	.70
	stools/poops at least once per day (24 h).	.61
	likes to put fingers and/or toys in mouth.	.57
	sucks strong enough to get milk from the bottle.	.53
	sucks strong enough to get milk from the breast.	.42
	sleeps well lying flat on his/her back.	.39
*Energy & Physiologic Stability* 13 items Cronbach’s α .81	gets exhausted during eating and is not able to finish.	.67
	is exhausted after eating.	.56
	can only suck a few times before needing to take a break.	.55
	needs to be encouraged to keep eating (such as, by touching or talking).	.52
	needs tube feedings.	.50
	gets pale or blue color around lips when eating.	.48
	needs to rest during eating to catch his/her breath.	.47
	takes more than 30 min to eat (including rest/burping periods).	.47
	breathes faster or harder when eating.	.45
	holds breath when eating.	.44
	eats more than 12 times per day (24 h).	.37
	wants to eat again within an hour after feeding.	.30
	sweats/gets clammy when eating.	.30
*Sensory Responsiveness* 7 items Cronbach’s α .77	chews or bites on the nipple (bottle) when he/she should be sucking.	.66
	will only eat if food (milk/formula/baby food) is a certain temperature.	.66
	will only eat from a specific kind of bottle/nipple.	.62
	will only take the bottle from specific people (such as, by mom).	.57
	refuses the bottle before having eaten enough (such as, turns head, pushes bottle away, pushes nipple out of mouth with tongue).	.54
	needs help latching on to the bottle.	.45
	will only eat if fed in a certain way (for example, in a certain chair, or held upright).	.34
*Feeding Flexibility* 10 items Cronbach’s α .79	is happy to eat from either the bottle or breast.	.66
	will eat expressed breastmilk that has been frozen and reheated.	.65
	needs help latching on to the breast (for example, needs a nipple shield or positioning help).	.59
	refuses the breast before having eaten enough (such as, turns head, pushes breast away, pushes nipple out of mouth with tongue).	.57
	chews or bites on the nipple (breast) when he/she should be sucking.	.56
	has a hard time handling how fast milk comes out of the breast (for example, chokes, coughs, gags, or pulls off the breast).	.56
	prefers bottle-feeding over breastfeeding.	.55
	prefers breastfeeding over bottle-feeding.	.51
	needs a bottle after breastfeeding.	.50
	gags on the breast.	.46

### Internal consistency reliability

All five subscales had acceptable internal consistency reliability: *Gastrointestinal Tract Function* subscale (Cronbach’s α = .91), *Infant Regulation* (Cronbach’s α = .86), *Energy & Physiologic Stability* (Cronbach’s α = .81), *Sensory Responsiveness* (Cronbach’s α = .77), and *Feeding Flexibility* (Cronbach’s α = .79). There were two items in the *Infant Regulation* subscale that, if deleted, would cause the Cronbach’s α to increase from .86 to .87. These two items were determined to be important items, had item-total correlations greater than .3 (i.e., acceptable), and the increase in Cronbach’s α was deemed insignificant, so the items were kept. One item on the *Energy & Physiologic Stability* subscale would cause the Cronbach’s α for that subscale to increase from .81 to .817, but this item also had item-total correlation greater than .3; this increase in Cronbach’s α was also deemed insignificant, so the item was kept. No other items would increase the Cronbach’s α if deleted and all items had item-total correlations > .3. The internal consistency reliability of the full 68-item scale was acceptable (Cronbach’s α = .88).

### Temporal stability

All subscale scores were highly and significantly correlated between the initial survey and the retest survey: *Gastrointestinal Tract Function* subscale (*n* = 50; *r* = .84, *p* < .001), *Infant Regulation* (*n* = 52; *r* = .82, *p* < .001)), *Energy & Physiologic Stability* (*n* = 52; *r* = .88, *p* < .001), *Sensory Responsiveness* (*n* = 50; *r* = .77, *p* < .001), and *Feeding Flexibility* (*n* = 51; *r* = .81, *p* < .001). The NeoEAT – Mixed Feeding total score was also highly and significantly correlated between the initial survey and the retest survey (*n* = 43; *r* = .91, *p* < .001).

### Convergent validity

The NeoEAT – Mixed Feeding total score was moderately and significantly correlated with the IGERQ-R sum score (*r* = .57, *p* < .001) and the IGSQ sum score (*r* = .5, *p* < .001). Correlations between the NeoEAT – Mixed Feeding subscale scores, IGERQ-R, and IGSQ are presented on Table 5.

**Table 5:** Correlation between the NeoEAT-Mixed Feeding, IGERQ-R and IGSQ

NeoEAT-Mixed Feeding	IGERQ-R sum score (*n* = 601)	IGSQ sum score (*n* = 363)
Total Score	.57**	.50**
Subscale Scores		
*Gastrointestinal Tract Function*	.71**	.61**
*Infant Regulation*	−.08*	−.06
*Energy & Physiologic Stability*	.52**	.45**
*Sensory Responsiveness*	.23**	.23**
*Feeding Flexibility*	.08*	.06

Pearson product-moment correlations are presented as an *r* value

*Indicates that the correlation was statistically significant (two-tailed) at *p* < .05

**Indicates *p* < .001

*IGERQ-R* Infant Gastroesophageal Reflux Questionnaire – Revised, *IGSQ* Infant Gastrointestinal Symptoms Questionnaire

### Known-groups validity

**Fig. 2:**
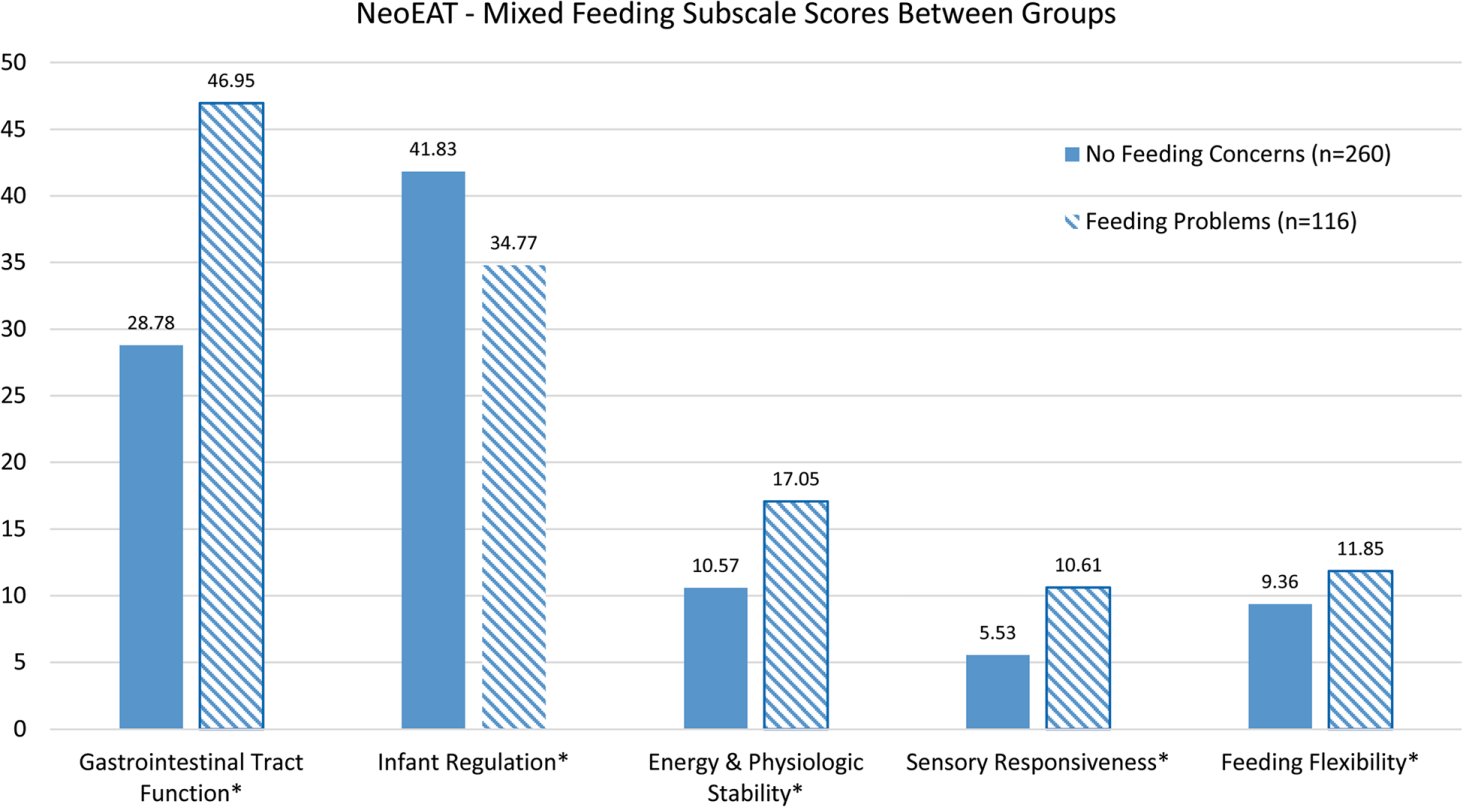
NeoEAT-Mixed Feeding subscale score differences between infants with feeding problems and infants with no feeding concerns. Note that high scores indicate more symptoms of problems in each subscale area. * Indificates *p* < .05. Infants in the “No Feeding Concerns” group had none of the following: history of preterm birth, genetic disorder, congenital heart disease, daily prescription medication use, developmental delay, diagnose or undiagnose feeding problem, feeding tube, structural abnormality of the face, mouth, or gastrointestinal tract, or difficulty with breast- or – bottle-feeding. Infants with a feeding problem were reported by parents as having a parent-identified feeding problem, a diagnose feeding problem, and/or need for a feeding tube

The NeoEAT – Mixed Feeding total score differentiated infants with problematic feeding (*M* = 121.23, *SD* = 33.64) from healthy infants without feeding concerns (*M* = 96.07, *SD* = 22.48; *t* [162.54] = − 7.36, *p* < .001). All five subscales also differentiated infants with feeding problems from those without feeding concerns (Fig. 2). Infants with feeding problems had significantly fewer symptoms of problems with *Infant Regulation* (*M* = 34.77, *SD* = 9.97) than infants without feeding concerns (*M* = 41.83, *SD* = 5.21; *t* [143.73] = 7.2, *p* < .001). Infants with feeding problems had significantly higher symptoms of problems on all other subscales compared to infants without feeding concerns: *Gastrointestinal Tract Function* (*t* [152.93] = − 8.73, *p* < .001), *Energy & Physiologic Stability* (*t* [153.6] = − 6.26, *p* < .001), *Sensory Responsiveness* (*t* [183.45] = − 6.91, *p* < .001), and *Feeding Flexibility* (*t* [184.53] = − 2.37, *p* = .02).

## Discussion

The NeoEAT – Mixed Feeding is a new, parent-report measure of symptoms of problematic feeding for infants who are feeding using a combination of breast- and bottle-feeding. The data presented in this paper reports on the item reduction strategy and exploratory factor analysis that determined the NeoEAT – Mixed Feeding is a 68-item measure with five subscales: *Gastrointestinal Tract Function*, *Infant Regulation*, *Energy & Physiologic Stability*, *Sensory Responsiveness*, and *Feeding Flexibility*. Psychometric testing results provide evidence that the NeoEAT – Mixed Feeding has acceptable internal consistency reliability, temporal stability, convergent validity, and known-groups validity (Table 6).

**Table 6:** Summary of the Properties of the NeoEAT – Mixed Feeding

Tool (Author)	Purpose & intended user	Target population	Items – number & constructs	Reliability			Validity	
				Internal consistency	Inter-rater	Test-retest	Content	Construct
Neonatal Eating Assessment Tool – Mixed Breast –and Bottle-Feeding (NeoEAT – Mixed Feeding)	Assess infant behavior during feeding in infants who are feeding at both breast and bottle; Parent	All infants birth to 7 months old, including those born healthy, full-term, preterm, and with any medical conditions	68 items; Infant regulation, gastrointestinal tract function, energy & physiologic stability, sensory responsiveness, and feeding flexibility	+	NR	+	+	+
				Internal consistency- Cronbach’s α = .88 for full scale, Cronbach’s α = .77–.91 for all subscales. Test-retest – *r* = .91 for full scale, *r* = .77–.88 for all subscales.			Content validity with 9 professionals – scale level CVI = .90 for clarity and .93 for relevance. Content validated with 19 parents using cognitive interviews [39]. Construct validity – Concurrent validity with the I-GERQ-R (*r* = .57*; p* < .001) and IGSQ (*r* = .5; *p* < .001). Known-groups validity- infants with feeding problems scored significantly higher than those without for full scale (*p* < .001) and all subscales (*p* < .05).	

+Indicates acceptable reliability/validity, − indicates unacceptable reliability/validity

*NR* Not Reported, *CVI* Content Validity Index, *I-GERQ-R* Infant Gastroesophageal Reflux Questionnaire – Revised, *IGSQ* Infant Gastrointestinal Symptoms Questionnaire. Acceptable reliability and/or validity defined as Cronbach’s α > .7 [36] or correlation coefficient (*r*) > .4 [38]

The results of the convergent validity testing were not all statistically significant, but this was to be expected given the constructs measured by the different parent-report measures used. The IGERQ-R, a measure of symptoms of gastroesophageal reflux, and IGSQ, a measure of symptoms of gastrointestinal distress, were found to be highly correlated, as expected, with the *Gastrointestinal Tract Function* subscale. These measures were not found to be highly correlated with the *Infant Regulation* or *Feeding Flexibility* subscales, which was expected because the IGERQ-R and IGSQ do not intend to measure feeding behaviors.

When the NeoEAT – Mixed Feeding scores were compared between a group of healthy infants with no feeding concerns and a group of infants with problematic feeding, the infants with problematic feeding were found to have higher (i.e., worse) NeoEAT – Mixed Feeding scores for the total score and the *Gastrointestinal Tract Function*, *Energy & Physiologic Stability*, *Sensory Responsiveness*, and *Feeding Flexibility* subscales; these findings were consistent with what was expected. However, the infants with problematic feeding were found to have significantly lower (i.e., better) subscale scores for the *Infant Regulation* subscale compared to healthy infants with no feeding concerns. The reason for this unexpected finding is unclear. There were ten infants in the problematic feeding group that currently had a feeding tube. It may have been that having a feeding tube changed the way these parents responded to questions like “eats enough to have a least 5 wet diapers per day” or “is satisfied after eating.” Alternatively, or concurrently, other studies have found that many healthy infants in the first 6 months of life struggle with self-regulatory behaviors [50]. The results of the known-groups comparison for infant regulation may be a reflection of the larger sample size of infants with no feeding concerns compared to those with feeding concerns and a high level of symptoms of difficulty with regulation even in healthy infants with no feeding concerns. The construct of infant regulation between infants with feeding concerns and no feeding concerns requires further investigation.

### Limitations

The primary limitations of this study were that it was conducted using an online survey and the respondents were primarily White mothers from two-parent families. The intended sample for this study was a large, geographically and racio-ethnically diverse sample representing data from both healthy infants and infants with medical complexity that impacted their feeding behaviors. An online survey was the best mechanism for obtaining this type of sample, but the inherent risks of online survey data collection are acknowledged and multiple strategies were instituted to respond appropriately to these risks.

Despite our varied recruitment methods, the sample was primarily mothers, which was expected since, in the United States where the majority of the sample was from, mothers tend to be the primary caregiver of young infants, and the sample was 65.7% White. According to the United States Census data from 2018, 76.6% of the United States population identified as White [51], so the proportion of the sample that identified as White was less than that in the general United States population. The proportion of the sample that identified as Hispanic, Black, and Asian was less than the general United States population, but the proportion of the sample that identified as being more than one race (14.8%) was considerably higher than that in the general United States population (2.7%) [51]. Although the sample being predominantly White was consistent with the population sampled, this may limit the generalizability of the findings. Future studies of the reliability and validity of the NeoEAT – Mixed Feeding should aim to include a more racio-ethnically diverse samples.

### Future directions

The next step for the NeoEAT – Mixed Feeding is to establish norm-reference values for the scores based on a large sample of healthy, typically feeding infants; these reference values will facilitate interpretation of scores relative to the range of typical feeding behaviors in young infants. Sensitivity and specificity of the cut-off scores developed from the norm-reference sample will need to be tested. Validation of the NeoEAT – Mixed Feeding scores against clinician feeding observation will provide further support for the use of the tool in clinical practice. A shorter, screening version of the NeoEAT – Mixed Feeding is under development.

## Conclusions

The NeoEAT – Mixed Feeding is the first parent-report measure of symptoms of problematic feeding with evidence of validity and reliability that can be used with infants who are doing a combination of breast- and bottle-feeding. The NeoEAT – Mixed Feeding can now be used in clinical practice and research to identify infants with problematic feeding and monitor response to treatment. Additionally, the subscales of the NeoEAT – Mixed Feeding may help to guide clinicians in understanding the underlying etiologies of the infant’s feeding difficulties and personalize treatment and referral decisions to best meet the infant’s needs.

## Data Availability

The dataset analyzed during the current study may be available from the corresponding author on reasonable request.

## Abbreviations

CTSA: Clinical translational science award program
EFS: Early feeding skills assessment
GERD: Gastroesophageal reflux disease
HaPI: Health and Psychosocial Instruments database
ICFQ: Infant and child feeding questionnaire
I-GERQ-R: Infant gastroesophageal reflux questionnaire—revised
IGSQ: Infant Gastrointestinal Symptoms Questionnaire
KMO: The kaiser-meyer-olkin statistic
*M*: Mean
NEO: Neonatal eating outcome assessment
NeoEAT: Neonatal Eating Assessment Tool
NeoEAT-Bottle-feeding: Neonatal Eating Assessment Tool- Bottle-feeding
NeoEAT-Breastfeeding: Neonatal Eating Assessment Tool- Breastfeeding
NeoEAT—Mixed Feeding: Neonatal eating assessment tool- mixed breastfeeding and bottle-feeding
NOMAS: Neonatal Oral Motor Assessment Scale
*SD*: Standard deviation
